# An innovative approach to near-infrared spectroscopy using a standard mobile device and its clinical application in the real-time visualization of peripheral veins

**DOI:** 10.1186/s12911-014-0100-z

**Published:** 2014-11-25

**Authors:** Simon Juric, Borut Zalik

**Affiliations:** Advanced ICT Research Group (AIRG), Farmadent Pharm, Minařikova ulica 6, 2000 Maribor, Slovenia; Laboratory of Geometric Modeling and Multimedia Algorithms, University of Maribor, Faculty of Electrical Engineering and Computer Science, Smetanova 17, 2000 Maribor, Slovenia

**Keywords:** Mobile applications, Spectroscopy, Near-infrared, Vascular access devices, Health education, Feasibility studies

## Abstract

**Background:**

Excessive venipunctures are a significant problem both in emergency rooms and during hospital stays. Near-infrared (NIR) illumination devices improve venipuncture success rate but their usage is limited by their availability and economic cost. The objectives of this study were to develop a low-cost NIR spectroscopy prototype from a standard mobile device, to evaluate its efficacy and acceptance as an educational tool, and in a clinical setting.

**Methods:**

Through a user-centric design process a prototype device was developed. Its educational efficacy was evaluated through a non-invasive, observational study (20 student clinicians, 25 subjects) and its acceptance was assessed using quantitative and qualitative analysis. A smaller clinical trial was performed by a group of 4 medical professionals over a period of 6 weeks that involved 64 patients.

**Results:**

The prototype enables real-time visualization of peripheral veins on a variety of Android-based devices. The prototype was 35.2% more successful in visualizing and locating veins (n = 500 attempts) than the nursing students. The acceptance assessment revealed high perception of usefulness, satisfaction, and ease of use. In the clinical trial, 1.6 (SD 1.3) additional veins per patient were identified compared with the traditional visualization methods.

**Conclusions:**

To the best of our knowledge this is the first study that describes the design, feasibility and application of an NIR spectroscopy prototype developed on a standard mobile device.

**Electronic supplementary material:**

The online version of this article (doi:10.1186/s12911-014-0100-z) contains supplementary material, which is available to authorized users.

## Background

### Introduction

Venipuncture is the process of obtaining intravenous access and is an everyday invasive medical procedure. Among patients admitted into hospital wards, the prevalence of a peripheral venous access line is as high as 80% depending on the condition of the patient and the location of the hospital [1]. Although a peripheral vein can be accessed in a single attempt, in a substantial number of patients the attending nurse needs between 2 and 10 attempts to insert the needle successfully [2]. The main causes for multiple attempts are: lack of adequate venipuncture skills, lack of adequate care and maintenance [3,4], or a medical situation regarded as a peripheral difficult venous access [5]. Excessive venipunctures are a significant problem (time- and resource-consuming events) in emergency rooms and during a hospital stay. Several approaches have been developed to improve venipuncture success rate [1]. There are four main strategies: (i) manual procedures with the aid of chemicals, but these are not suitable for children and are not effective on people with dark skin, (ii) the use of ultrasound-guided procedures, which have the disadvantage of the need for additional trained staff and expensive equipment, (iii) the use of secondary light sources, which requires a darkened room and can cause burns, and (iv) the visualization of the venous system by means of near infrared (NIR) spectroscopy [6].

Veins contain deoxygenated hemoglobin-rich blood that almost completely absorbs light at near-infrared wavelengths (740 nm–760 nm) at a distance of up to several centimeters. The situation is the reverse for the oxygen-rich arterial blood supply. NIR spectroscopy takes advantage of this differential absorption to clearly distinguish the veins from the arteries and the surrounding tissue [7].

There are now several medical devices that utilize NIR to aid vascular access procedures; e.g., VeinViewer® [8], AccuVein® [9], Veinsite® [10], and VascuLuminator [11]. These commercial NIR devices provide a valuable clinical function but their accessibility is limited by their cost. To overcome this limitation we propose a mobile health (m-health) solution.

M-health is an emerging field of practical solutions for improving healthcare using mobile devices coupled with new information and communication technologies to lower costs, enhance patient safety and strengthen the quality of care in diverse settings and medical specialties [12-16]. A recent report affirms that there will be more than 500 million active m-health users by 2015 [17].

The first wave of m-health applications solved technologically simple problems (e.g., coordination of appointments, reminders, self-management of long-term chronic diseases and diet/weight counselling). The new generation of m-health applications target a broad range clinical and management challenges. There have been breakthroughs in using mobile devices as diagnostic instruments, e.g., in ophthalmology [18], neurosurgery [19], and Doppler ultrasound [20]. For this new generation of applications the term mobile medical apps was introduced, which are defined as “medical devices that are mobile apps, meet the definition of a medical device and are an accessory to a regulated medical device or transform a mobile platform into a regulated medical device” [21].

### Objectives

This paper describes the design, development and initial evaluation of a mobile medical app called mVeinVision that aims to assist and improve venipuncture. The app is implemented on a standard mobile device, is simple to use and intended as a low-cost alternative to commercial NIR devices. For details of how the conceptual design of the current prototype evolved from a review of clinical studies evaluating NIR spectroscopy, the interested reader is referred to Juric et al. [22].

## Methods

### Prototype development

An NIR spectroscope is a device that has an NIR light source on one side and an NIR sensitive camera on the other that is able to capture the surface illuminated by the light source. Some post-processing of the NIR image is usually required for clinical usage. The main objective was to integrate a light source, camera and image processing algorithms to produce a novel mobile application.

The primary design objectives of the prototype were that: (i) it uses the native functionality of the device as much as possible, (ii) limits additional hardware to low-cost peripherals that are easily installable, (iii) is powered only by the device itself, (iv) is simple to use, and (v) visualizes the veins in real time as clearly as the commercial devices.

Android operating system was chosen as the development platform for the prototype. Instead of using one of the special NIR sensitive cameras employed in commercial systems, to keep to the device economical a standard external Universal Serial Bus (USB) camera was modified and used. This involved removing the built-in filter (hot mirror), which blocks wavelengths above 720 nm (i.e., those in the NIR spectrum), and replacing it with a pass-through filter made from an exposed and developed empty 35 mm camera film [23]. The focus was also readjusted. This approach has shown promising results in other low-cost vein detection applications [24-26]. The camera is connected to the mobile device using a USB On-The-Go (OTG) cable and the in-house developed controlling software (EXCAM: External Camera Management). A set of low-energy NIR light-emitting diodes (LEDs) are used in order to provide the uniform NIR illumination (NIA: NIR Illumination Accessory). The software also contains an image processing component (NIP: NIR Image Processing) and a graphical user interface (UI). Safety risks of the prototype were inherently minimized in the design (see Additional file 1).

**Figure 1:**
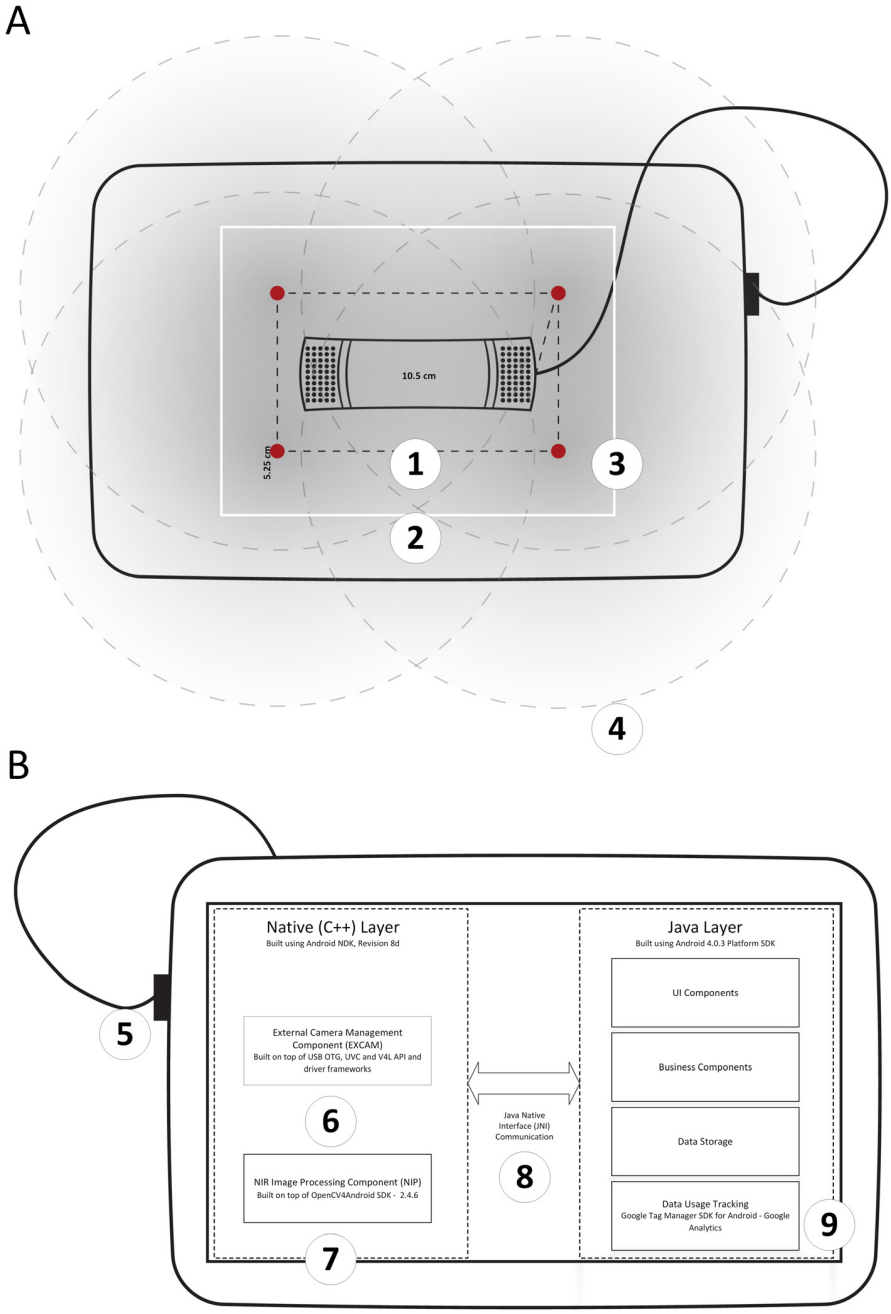
**Architecture of mVeinVision device, comprised of (A) external (NIR modified) USB Camera and NIR illumination accessory (NIA) architecture and layout – embedded into a low-cost silicon case on the back side of the device, and (B) high level architecture of the core Android application component of mVeinVision. (1)** USB camera converted to be NIR sensitive with readjusted focus and connected to the device through a **(2)** Micro-B USB Host OTG Cable. The camera is attached using a self-adhesive Velcro strip. **(3)** NIA consisting of 4 rectangular connected IR high intensity LEDs (OIS-330-740-X-T, peak wavelength of 740 nm, forward current of 30 mA) embedded into a back cover silicon case and connected to the power supply of the camera via a USB 2.0 cable (5 V, 500 mA). **(4)** Radial NIR illumination distribution of 1 LED with the most luminous intensity around the center and dropping along the radius. **(5)** The field of view of the camera which corresponds to the most illuminated area by NIA. **(6)** External Camera Management (EXCAM) software library that controls external USB camera. The library supports video capture in native camera video resolution. **(7)** NIR Image Processing (NIP) software library. **(8)** User Interface (UI) and background processing components. **(9)** Communication layer for managed code (Java) that permits interaction with the native code (C/C++).

**Figure 2:**
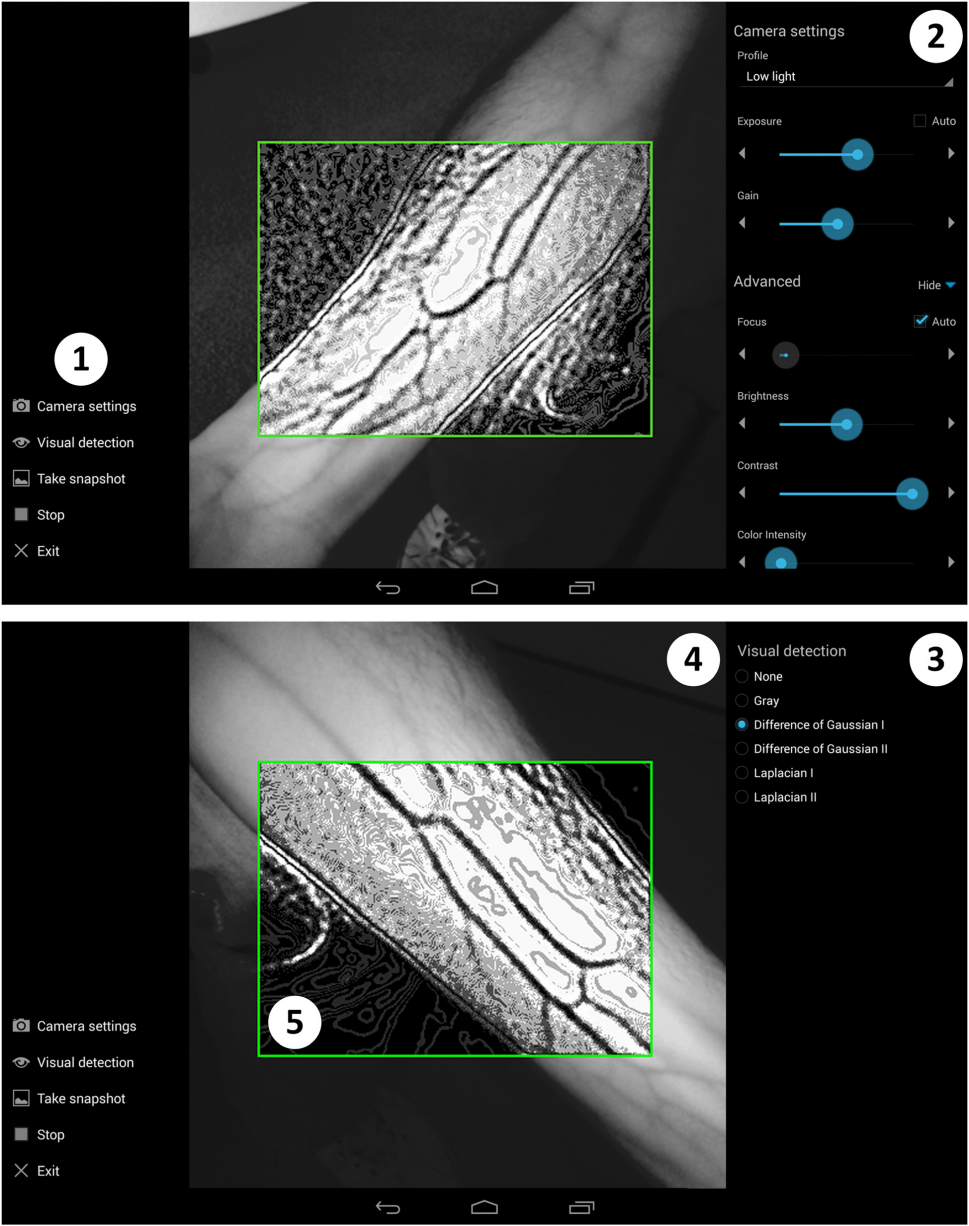
**User interface of mVeinVision, which is shown for the (2) camera settings and (3) visual detection functions.** The contextual menus are displayed through the corresponding option in **(1)**. **(2)** Contains the controls through which a user can manually adjust the camera settings in relation to the environmental conditions (e.g., lighting) in order to improve vein visualization quality. The optimal values of these settings were experimentally determined (from user feedback and the most common environmental conditions) and grouped into 5 profiles (Low light, Medium light, Very bright, LED profile and Custom). **(3)** Additional visualization controls corresponding to image processing strategies presented in Figure 1-B. **(4)** Captured NIR video preview with the applied image processing in the **(5)** region of interest.

The architecture of the prototype is shown in Figure 1. For efficiency, the camera settings (e.g., gain, exposure and contrast) are optimized before image capture (EXCAM), minimizing the opportunity for post-processing artifacts. However, some image enhancement is needed for visualization of the veins (NIP) because of the high contrast that the NIR illumination produces. This involves the application of difference of Gaussians and Laplacian operators for edge detection and feature smoothing (see Additional file 2). The real-time vein detection is displayed at 12 frames per second (Figure 2).

The hardware components required to deploy the mVeinVision app on an Android-based device cost 30–80 USD, depending on the camera model.

#### Ethical aspects

The evaluation part of the study was assessed by the FERI institutional and ethics review board (University of Maribor – reference number: 3/2188-ŽĐ/2013). The board concluded the study exempt from further ethical approval because: (i) it was a preliminary non-invasive, observational feasibility study, (ii) no identifying information was collected from the participants (which were also anonymized), (iii) it was supervised by health care professionals, and (iv) no further intervention on participants was planned based on the results of the evaluation. All the participants (or their legal guardians) gave written informed consent to participate in all stages of the evaluation studies.

### Educational application

#### Recruitment

**Figure 3:**
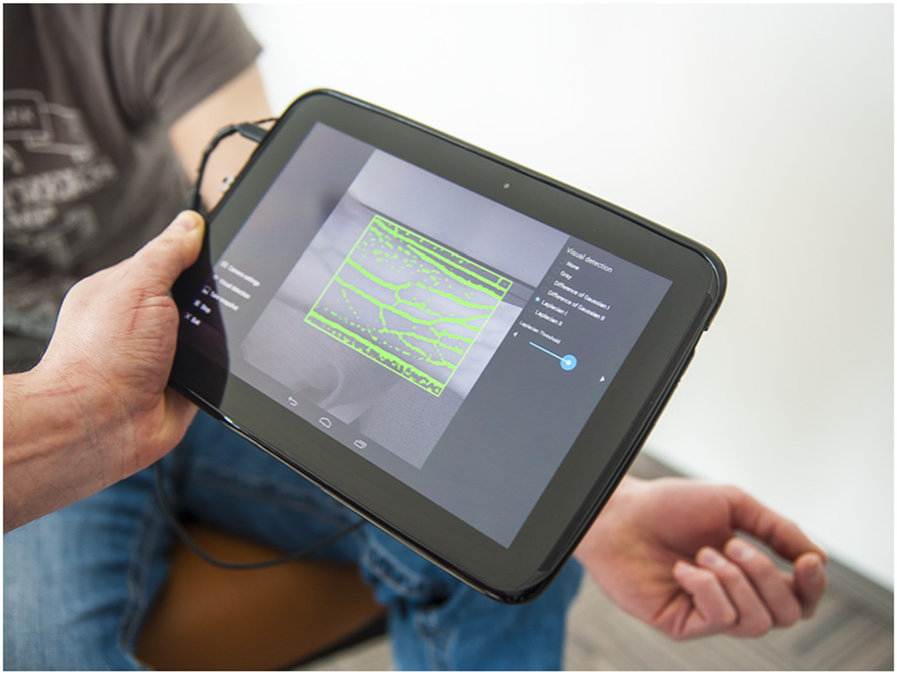
**Prototype during the suitability assessment (pre-screening).** An example of sufficient visibility of the subcutaneous veins on the inner elbow with additional visualization of the vein pattern using the Laplacian I processing.

43 trainee clinicians and 72 subjects were recruited. The eligibility criteria for the clinicians to enroll were being a nursing student, having completed a theoretical and supervised practical program in phlebotomy that included venipuncture, and being an experienced smartphone user. Two medical professionals and one smartphone developer conducted short interviews with the trainee clinicians to ensure that they had a contextual understanding of the research topic, were aware of the negative impacts of unsuccessful venipuncture, were willing to learn professional skills and would be available when required. On this basis a final group of 20 nursing students were selected from the original 43 enrolled. The eligibility criterion for the subjects to enroll was low or zero visibility of subcutaneous veins on the inner elbow by the naked eye. To participate in the study the subjects also needed sufficient visibility of these veins with the prototype device (Figure 3). This was determined by the two medical professionals, reducing the number of subjects from 72 to 25. The two medical professionals supervised the study and all of the participants (n = 47) willingly agreed to take part.

#### Vein detection

The first aim was to evaluate the prototype application in the visualization and location of veins by comparing its efficacy with the nursing students’ non-invasive skills (e.g., visual check, palpation). The device was not presented formally to the students prior to this exercise. The students were asked to select a point on the inner elbow (e.g., the antecubital fossa) of the subject where they thought it was most convenient for venipuncture, and then they marked the location with a pen. This mark was defined as a venipuncture attempt. The time needed to perform the procedure was not taken into account although there was a maximum of 2 min available. The marked point was then visualized with the prototype application to check if there was a vein underneath. A positive match was defined as a successful attempt. All attempts were reviewed by the supervising professionals. The trainee clinicians repeated this procedure on each of the subjects (n = 500 attempts).

#### Usage evaluation

At the beginning of the second exercise each student received a 3-hour presentation of the prototype along with the practical training needed for its stand-alone use. After the demonstration, the students were given an opportunity to further evaluate the application for a period of 2 weeks in exchange for a follow-up interview and a questionnaire commenting on their experience. After the pilot usage period, the degree of acceptance among the students was evaluated from the recorded usage data and a Likert-scale questionnaire that estimated: (i) perceived usefulness, (ii) satisfaction, (iii) ease of use, and (iv) previous knowledge. All statistical analyses were performed with IBM SPSS Statistics 21.0 (IBM Corporation, Armonk, NY, USA).

### Clinical application

#### Recruitment

Four medical professionals from the University Medical Centre Maribor (Slovenia) agreed to participate. They consisted of 1 physician, 3 experienced nurses and only patients who agreed to participate were enrolled. During the observational evaluation mVeinVision was used on 64 anonymized patients, 27 of which were classified as difficult cases based on advanced age, drug abuse, obesity, poor vein quality or had previous history of difficult vein access owing to their medical conditions.

#### Vein detection

Two mVeinVision enhanced devices (Samsung GALAXY Nexus) were used for a period of 6 weeks at the University Medical Centre Maribor. The aim was to evaluate the efficacy of the prototype on improving the visibility of peripheral veins on real patients, when compared with the traditional methods. The 4 medical professionals were instructed on how to use the device and received a 1-hour training session prior to clinical usage.

#### Usage evaluation

After the 6-week trial period, the clinicians were asked to complete a questionnaire to assess the general usability of the device in a clinical setting. The questionnaire was based on a system usability scale (SUS), comprising 10 questions with 5 response options (Likert scale). The SUS is an industry standard for reliable and valid assessment of perceived usability [27]. A freeware web-based office suite (Google Docs) was used for tracking the evaluation results and the self-report SUS questionnaire.

## Results

**Figure 4:**
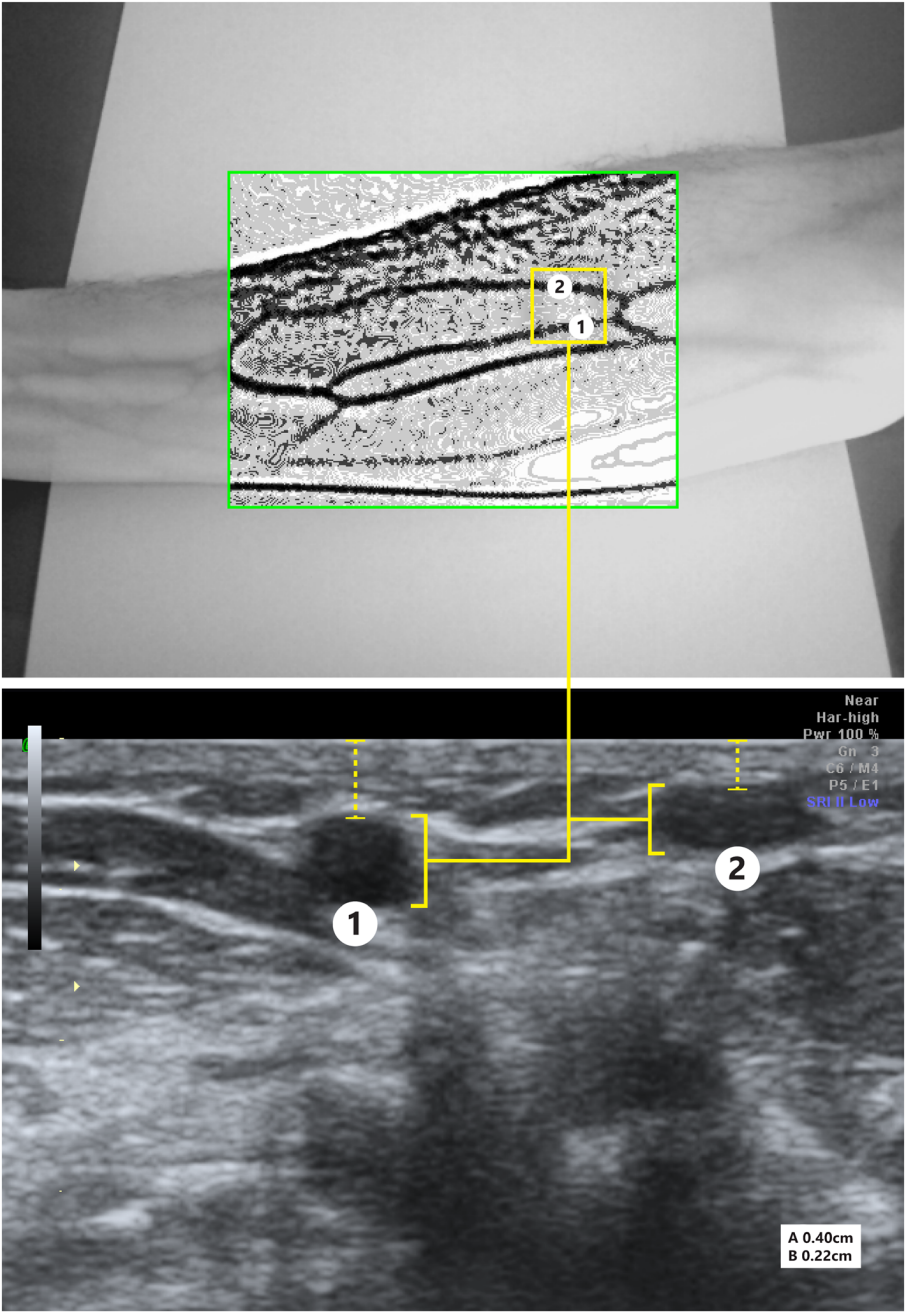
**An image from the prototype (above) and an associated cross-sectional ultrasound image confirming the presence of a pair of veins below the skin surface.** A (0.40 cm) and B (0.22 cm) represent the depth of the veins as identified with the ultrasound device.

To establish the accuracy of the prototype for use as a scientific instrument in the educational and clinical applications, it was priorly tested on 5 subjects where the real-time preview and captured images were compared with those from an ultrasound-scanning device (ACUSON Sequoia 512 Ultrasound System, Siemens Medical Solutions USA, Inc., Malvern, PA, United States). The prototype was calibrated prior to use. All experiments were performed during the daytime using a combination of artificial and ambient natural light. The test area was the forearm section from the antecubital fossa to the wrist. First, the prototype was used to visualize the subject’s veins, two capture images were recorded (a raw contrast-enhanced image and a post-processed image), and then the skin surface was segmented into rectangular areas and marked with a pen to indicate the venous network. Next, the subject’s forearm was scanned with the ultrasound. The probe traversed each bounding box producing cross-sectional images of the veins. Figure 4 shows an image from the prototype (above) and the corresponding ultrasound scan for comparison (below). On average the prototype was able to accurately visualize veins that were up to 4.8 mm (SD 0.7 mm) below the skin surface.

### Educational application

Table 1 shows the results of the 500 supervised attempts collectively made by the 20 student clinicians to locate a site for venipuncture. The veins of all the patients (n = 25) were clearly visible with the prototype. A failed attempt was thus indicated by a mismatch of the marker location with the venous network visualized with the prototype, and subsequently confirmed by the supervising professionals. The global failure rate was 35.2% (176/500).

**Table 1:** **Failure rate of student clinicians in locating the veins from 500 attempts, classified by Body Mass Index (BMI) of the subject**

	**Failure/Attempts**	**Failure rate (%)**
BMI 1	23/80	28.8
BMI 2	119/360	33.0
BMI 3	34/60	56.7
Total	176/500	35.2

The results of the user acceptance assessment following 2 weeks of unsupervised usage are listed in Table 2. The quantitative usage data that were recorded are summarized in Table 3. On average, the student clinicians used the prototype 1.3 (SD 1.7) times daily. The high scores for Likert scale items A, B and C indicate that the users generally found the device useful, easy to use and were satisfied with it. These are particularly favorable outcomes from a group of users that had relatively little previous knowledge of vein imaging devices, indicated by the low score of questionnaire item D.

The results of the user acceptance assessment following 2 weeks of unsupervised usage are listed in Table 2. The quantitative usage data that were recorded are summarized in Table 3. On average, the student clinicians used the prototype 1.3 (SD 1.7) times daily. The high scores for Likert scale items A, B and C indicate that the users generally found the device useful, easy to use and were satisfied with it. These are particularly favorable outcomes from a group of users that had relatively little previous knowledge of vein imaging devices, indicated by the low score of questionnaire item D.

**Table 2:** **Questionnaire responses of student clinicians to evaluate usability**

	**Likert scale items**			
**Nursing student**	**A**	**B**	**C**	**D**
1	5	4	4	2
2	5	5	4	1
3	5	4	5	1
4	4	5	5	2
5	5	4	4	3
6	4	4	4	2
7	5	5	4	1
8	5	4	4	1
9	3	4	5	1
10	5	4	4	3
11	5	5	4	2
12	4	4	5	1
13	4	4	4	1
14	4	4	5	2
15	5	5	4	3
16	4	4	4	2
17	4	5	4	1
18	3	3	4	3
19	5	5	3	2
20	5	5	4	1
Mean	4.45	4.35	4.20	1.75
SD	0.686	0.587	0.523	0.786
Mode	5	4	4	1

Questions: (A) ‘The prototype is useful’ , (B) ‘I am satisfied with the prototype’ , (C) ‘The prototype is easy to use’ , and (D) ‘I had previous knowledge about vein imaging devices’.

**Table 3:** **Reports of user events which were captured over a 2-week period (n = 20 users)**

**Variable**	**Value**
**Measurement Metric: Session**	
Total number of sessions	497
Average session duration (hh:mm:ss)	00:08:52
Longest session duration	00:36:29
Most used screen (displayed controls)	Visual detection
**Measurement Metric: Events**	
Total number of manual camera settings adjustments	12,475
Three most used camera settings (descending based on the total number of usage)	Exposure, Contrast, Gain
Two most selected camera profiles (descending)	Low light, LED profile
Most used visualization mode (algorithm)	Difference of Gaussians I
Number of snapshots (captured images from real-time video preview)	286
Number of snapshots which contain veins (analyzed after the pilot period)	219
Average frame frequency (Hz)	12.21

Terminology: a ‘Session’ represents a single period of user interaction within the application, ‘Screens’ represent content which users are viewing within the application and ‘Events’ represent a way to collect data about a user’s specific engagement with interactive content (e.g., button presses).

### Clinical application

The number of additional veins identified per patient with the aid of mVeinVision was 1.6 (SD 1.3) compared with the traditional visualization methods. In the subgroup of 27 difficult cases, 2.4 (SD 1.8) additional veins per patient were identified.

The mean SUS score for all medical professionals (n = 4) was 76.75 (SD 8.21). According to Bangor et al. [28], based on nearly 10 years of empirical evaluation of the SUS, an SUS score above 70 is acceptable, less than 50 should be a cause of significant concern, while scores between 50 and 70 are marginally acceptable. Thus for the small sample size, the SUS scores were favorable.

## Discussion

To the best of our knowledge, this is the first study to evaluate a low-cost mobile medical app—developed on a standard mobile device—as an educational/training tool to assist nursing students in venipuncture. The prototype provides real-time visualization of peripheral veins using near-infrared videography on mobile devices supporting the Android operating system (versions 4.0.3 and higher).

Previous investigators have demonstrated the feasibility of low-cost vein detection systems within a research environment [24-26,29-32]. On this basis, the prototype was developed as a cost-effective replacement for commercial NIR devices and was tested in clinical setting. Moreover, this study demonstrated the utility of the prototype as an educational tool to help develop nursing students’ venipuncture skills. This is in line with current trends to integrate handheld technology into nursing training (e.g., at Yale and Harvard [33,34]).

In three subjects in the educational study, the prototype was unable to visualize any veins. Two subjects were obese (BMI = 31.4 and 32.7) thus the problem was related to the NIR penetration depth and camera sensitivity. On the third subject the intended venipuncture site was obscured by hair on the inner elbow. These three subjects were excluded from the study. However, similar problems have been encountered in clinical studies of commercial devices [35].

## Conclusions

This paper presents a novel low-cost solution for improving the success rate of vein location in venipuncture. Although the number of participants in these studies was relatively small, the preliminary evaluations suggest that the prototype device has potential utility as a learning aid in the medical curriculum and as a tool for venipuncture in hospitals, particularly with patients who are known to be difficult cases. This study serves as a scientific reference for future investigations of the device involving larger numbers of participants.

The mVeinVision software and the supporting documentation are available online [36].

## Additional files

Additional file 1:
**Safety evaluation of mVeinVision.**
Additional file 2:
**Visualization and image processing workflow of mVeinVision.**

## Abbreviations

BMI: Body mass index
EXCAM: External Camera Management Component
FDA: Food and Drug Administration
LED: Light-emitting diode
NIA: Near-infrared illumination accessory
NIP: Near-infrared Image Processing
NIR: Near-infrared
OTG: On-the-go
UI: User interface
USB: Universal serial bus
SD: Standard deviation
