# Supplementary material for: An innovative approach to near-infrared spectroscopy using a standard mobile device and its clinical application in the real-time visualization of peripheral veins
Source: BMC Med Inform Decis Mak. 2014 Nov 25;14:100. doi: 10.1186/s12911-014-0100-z (PMC4251692; doi:10.1186/s12911-014-0100-z)
Supplement: Additional file 1: — Safety evaluation of mVeinVision. [file 12911_2014_100_MOESM1_ESM.docx]

**Safety evaluation**

Our prototype was made from a standard mobile device and (modified) USB camera. We considered the only potential safety risk to be skin burn injuries from the NIR light source—the NIR-emitting LEDs. It has been reported that their main source of heating is the semiconductor junction formed when the LED is pressed to the skin, which can cause temperature increases of up to 10°C for contact durations of 30 mins or more [1]. The absorption of NIR light by the skin may also cause temperature increases but only on the order of 0.5°C, as at the shorter wavelengths in this region (peak value 740 nm), the power is comparable with that of the NIR spectrum from sunlight [1]. Given that we operate our prototype and the NIR LED at a distance of least 30 cm from the patient’s body, avoiding skin-LED contact altogether, and that the LED is of the low-emitting variety (OIS-330-740-X-T, forward current of 30 mA), we deemed our prototype to pose no major risk to either the operator or the patient.

As previously mentioned, the device uses low-emitting NIR LEDs with a peak wavelength of 740 nm, rather than lasers, which pose a risk of tissue damage at wavelengths longer than 950 nm hence their utility in laser surgery [1]. The commercial Vein Viewer is one example of a vein visualization device that uses LEDs and has already been approved by the FDA. For examples of therapeutic devices that also demonstrate the safety of the LED light source technology, see Desmet et al [2]. In our prototype, the circuit does heat up when operating but as it is held at a distance of 30 cm from the patient, the likelihood of injury through contact is small.

**References**

1. Bozkurt, A & Onaral, B: **Safety assessment of near infrared light emitting diodes for diffuse optical measurements.** *Biomed Eng Online* 2004; 3:9. doi:10.1186/1475-925X-3-9.
2. Desmet KD, Paz DA, Corry JJ, et al.: **Clinical and experimental applications of NIR LED photobiomodulation**. *Photomed Laser Surg* 2006;24:121–128. PMID: 16706690
