# Supplementary material for: An innovative approach to near-infrared spectroscopy using a standard mobile device and its clinical application in the real-time visualization of peripheral veins
Source: BMC Med Inform Decis Mak. 2014 Nov 25;14:100. doi: 10.1186/s12911-014-0100-z (PMC4251692; doi:10.1186/s12911-014-0100-z)
Supplement: Additional file 2: — Visualization and image processing workflow of mVeinVision. [file 12911_2014_100_MOESM2_ESM.docx]

1. **Overview of the image processing workflow of mVeinVision.**


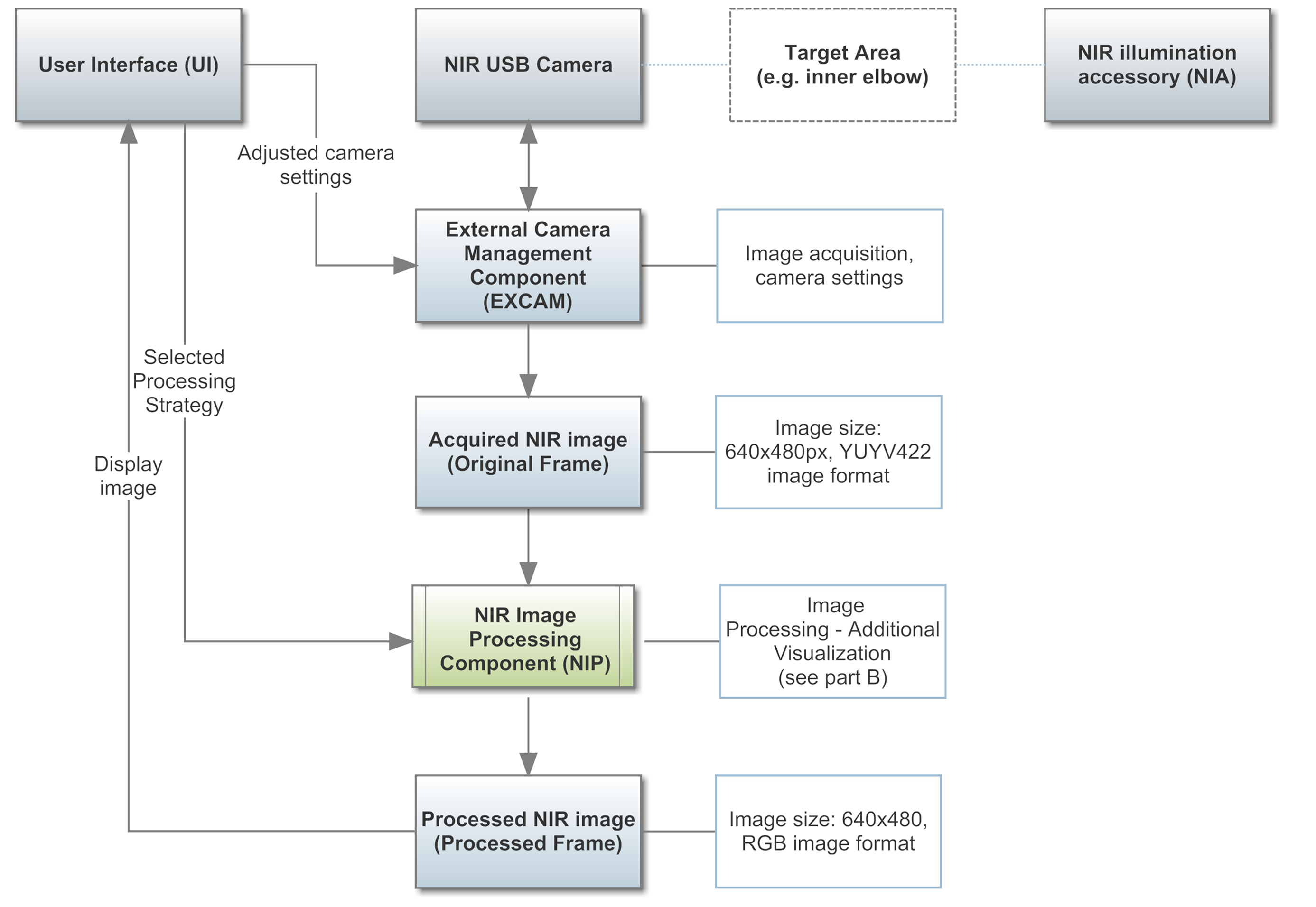


1. **Image processing workflow in NIR image processing (NIP), which is based on the user’s selection in the UI**


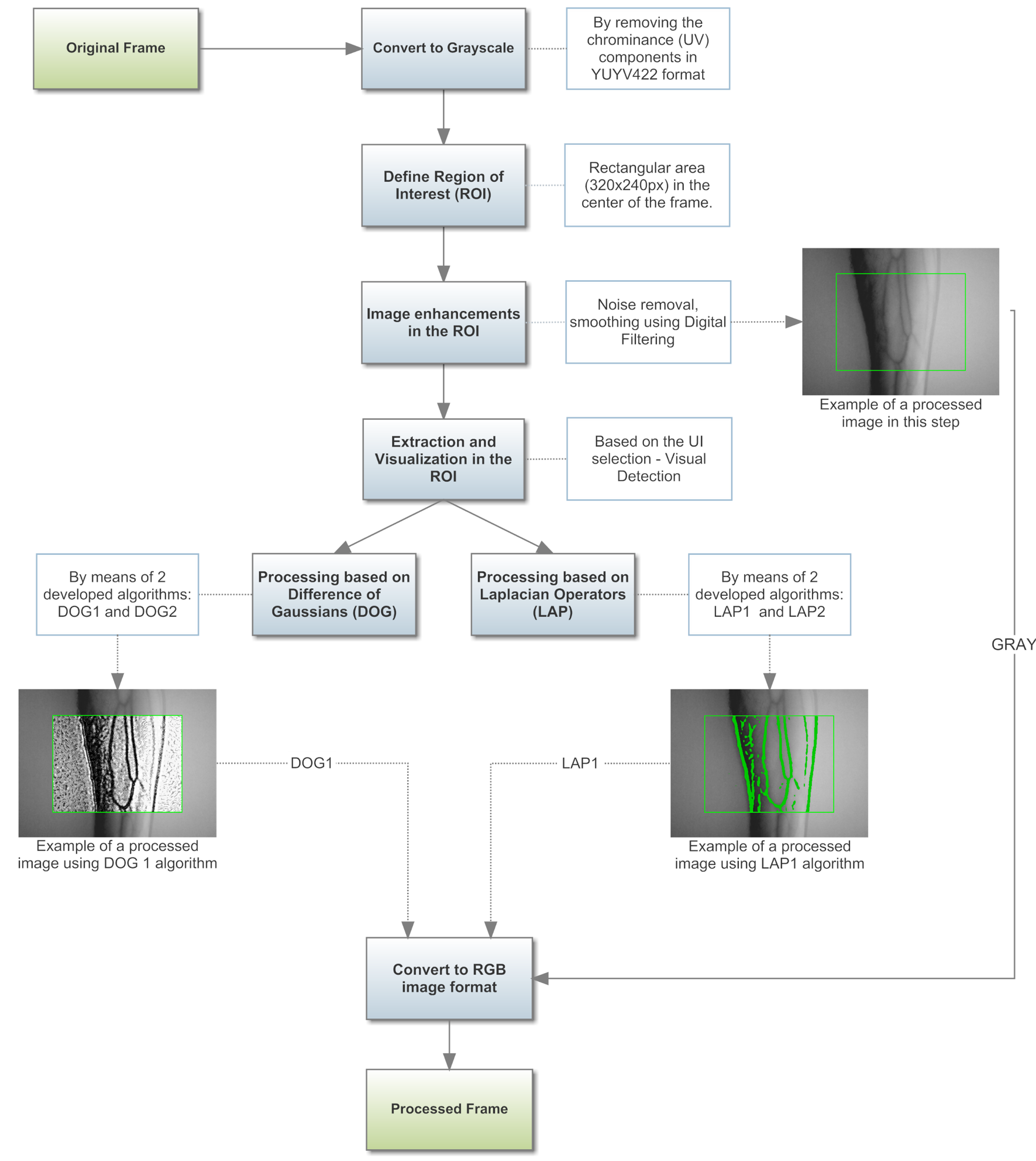


The following three options are available: (1) ‘None’, NIP returns the original acquired frame with no image processing, (2) ‘Gray’, the acquired frame is converted to grayscale, enhanced and returned, and (3) a group of modes which correspond to the four algorithms developed for the purpose of vein extraction and visualization (Difference of Gaussians I, II and Laplacian I, II). The output frame is converted to a Red Green Blue color space image.
